# Axial Crushing and Energy Absorption Integrated Design of Modular Filled Double-Hat Beam Composite Structures

**DOI:** 10.3390/ma17174302

**Published:** 2024-08-30

**Authors:** Xiaojian Yi, Lin Hu, Qiqi Li, Yong Tang

**Affiliations:** 1 School of Traffic and Transportation Engineering, Changsha University of Science and Technology, Changsha 410114, China; csust_yxj@163.com; 2 Hunan Province Key Laboratory of Safety Design and Reliability Technology for Engineering Vehicle, Changsha University of Science and Technology, Changsha 410114, China; hulin@csust.edu.cn (L.H.);; 3 School of Automotive and Mechanical Engineering, Changsha University of Science and Technology, Changsha 410114, China

**Keywords:** modular filled structures, double-hat beam, axial crushing response, energy absorption, composite structures, integrated design

## Abstract

In order to study the influence of modular filled and composite material forms on the axial crushing and energy absorption properties of structures, modular filled composite structures were constructed, and innovatively applied to the inner side of double-hat beam (DHB) structures in automobiles. The modular filled structures comprise hexagonal, quadrilateral, and triangular sections. By analyzing the collision performance of modular filled DHB structures, significant enhancements were observed in both the sectional characteristics and the specific Mean Crushing Force of modular filled DHBs compared to the conventional double-hat beam structure. These advancements notably improved the plastic deformation characteristics of the structures. Additionally, dynamic weightlessness experiments were conducted to validate the accuracy of the simulation model. Among the proposed schemes, namely QU-5, HE-5, and TR-5, notable improvements in crashworthiness were identified. Specifically, crashworthiness indicators increased by 32.54%, 78.9%, and 116.53%. Compared with other thin-walled structures, modular filled composite DHBs have advantages in axial crushing and energy absorption. By optimizing layout characteristics, the modular filled structures will achieve significant lightweight and energy absorption performance improvements. This work has clear reference value for automotive engineers and scholars to further explore the axial crash safety, platform modularization, and lightweight design of vehicles.

## 1. Introduction

Modular filled structures are endowed with specific functions and can achieve prominent interchangeability and universality. Through the combination of diverse modules, a range of products can be designed to meet the varied requirements of customers for application variability [1,2,3,4]. The characteristics of modules allow for their application across different products, thereby reducing the developmental costs and enhancing the performance of components. Modular structures [5,6,7,8] have been applied and validated in the fields of structural materials and functional materials. When studying fiber composites, researchers discovered that some composite materials exhibit negative Poisson’s ratio characteristics.

In the modular design and research of previous researchers, Herakovich et al. [9] proposed that different stacking sequences can be used to obtain modular fiber materials. Tsang et al. [10] designed a series of experiments to study a novel modular structure mimicking muscle tissue, and the results indicated outstanding energy absorption and mechanical performance for this structure. In the field of mechanics, Fan et al. [11] introduced a graded rectangular tube, where the Mean Crushing Force (MCF) gradually increases with the level of grading. Xu et al. [12] proposed a novel modular self-similar hexagonal tube, successfully enhancing energy absorption capacity and collision force efficiency. Li et al. [13] studied the axial crushing performance of graded metal thin-walled hexagonal tubes, revealing that the specific energy absorption (SEA) of graded metal thin-walled tubes is twice that of traditional single-cell tubes, suggesting that graded metal thin-walled tubes are more weight-efficient energy absorption devices. Liu et al. [14] investigated the in-plane and out-of-plane collision performance of modular structures obtained by introducing substructures at hexagon vertices, demonstrating that modular design significantly improves structural collision performance. Tao et al. [15] 3D printed a series of square modular structures, finding that these structures exhibit higher compression strength, collision force efficiency, and specific energy absorption compared to traditional single-material DHBs. Wang et al. [16] constructed a vertex-based and square thin-walled modular structure, and the results indicated that the vertex-based modular structure has stable compressibility.

In filling method research, current research into DHBs mainly focuses on utilizing plastic deformation to reduce collision kinetic energy, and its high strength, light weight, and excellent energy absorption performance make it widely applied in vehicle body structures [17,18,19,20]. In scenarios like frontal collisions and offset collisions, traditional DHB designs mainly cover structural optimization (including shape, size, topology, etc.) [21,22,23,24,25] and the application of composite materials, such as high-strength steel [26,27], aluminum–magnesium alloys [28,29], or composite materials [30,31,32]. These designs aim to ensure collision safety performance while achieving vehicle lightweighting [33,34]. In order to improve the spatial utilization of structures, researchers have conducted a series of studies on the methods and applications of foam aluminum, polyurethane, and honeycomb [8,35,36]. Sun et al. [37] proposed an innovative circular joint quadrilateral modular honeycomb structure, finding that this novel modular structure exhibits excellent energy absorption efficiency. Zorzetto et al. [38] successfully introduced structures with different Poisson’s ratios, generating a new composite material, and significantly improved the stiffness of the composite material by adding materials with negative Poisson’s ratios. Qi et al. [39] used foam aluminum to fill DHB to enhance its energy absorption efficiency under impact loads. Li et al. [40] investigated the axial crush characteristics of foam-aluminum-filled thin-walled square tubes, identifying adhesion as the main reason for enhancing the energy absorption characteristics of aluminum-filled thin-walled square tubes. A review of the current research status on the impact resistance of beam structures reveals that both modular methods and filling methods can effectively improve the impact resistance of modular structures [41,42].

Only a few studies focus on the fusion of the modular and filling methods. By reviewing the current research concerning tube beam structure crashworthiness, it has been concluded that both the modular and filling methodologies of modularity enhanced the structure crashworthiness. The novelty in this study lies in the innovative fusion of the modular and filling methods, entailing the arrangement and infusion of hexagonal, quadrilateral, and triangular aluminum grading structures within DHBs. Furthermore, a modular structural concept combining an aluminum modular structure with high-strength steel DHB was introduced to concurrently reduce structural mass (SM) and enhance axial energy absorption characteristics.

The specific structure of this article is as follows: Section 2 establishes the finite element model of DHB and validates the accuracy of the simulation model through drop weight tests. Section 3 introduces DHBs filled with three types. Section 4 and Section 5, respectively, discuss the influence of modular form and element size on the impact performance of the DHBs. Finally, Section 6 summarizes the main findings of this study.

## 2. FEA Models and Dynamic Experiments

In this chapter, we conducted finite element analysis of the DHB structure and compared it with dynamic experiments to verify its axial impact performance. Hypermesh and Ls-dyna are used in this section. Hypermesh is a software of Altair company in Troy, MI, USA. Ls-Dyna is a software of Ansys company in Canonsburg, PA, USA.

### 2.1. Finite Element Model of the DHBs

**Figure 1:**
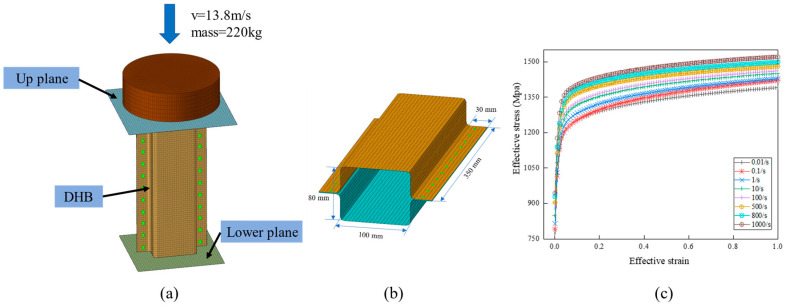
Finite element model of the DHB: (**a**) drop weight model, (**b**) geometric dimensions of the DHB, and (**c**) stress–strain curve of DP1180.

Figure 1a illustrates the finite element model of the DHB, comprising two U-shaped plates. The DHB’s base is anchored to a fully constrained rigid plate. In this model, a 220 kg weight impacts the DHB vertically at 13.8 m/s.

The contact between the DB and the hammer is based on penalty-based surface-to-surface contact, while the DB itself uses penalty-based single-surface contact. A tied-nodes-to-surface contact is employed between the two U-shaped plates and the spot welds. The specific parameter settings are as follows: The dynamic and static friction coefficients are set to 0.2. The material of the DB is DP1180, with Young’s modulus, Poisson’s ratio, and density values of 210 GPa, 0.3, and 7.85 × 103 kg/m^3^, respectively. The high-speed impact simulation of the DB considers the nonlinear stress–strain characteristics of DP1180 steel and its strain rate effect. The simulation analysis is conducted using the commercial software Hypermesh, version of 14.0.

Figure 1b shows the DHB’s geometric parameters and values. The shell element is integrated through the thickness direction using five integration points, providing accurate results for shell structures. The U-shaped plates in the simulation model are constructed using four-node fully integrated shell elements. These parameters include a length of 350 mm, section dimensions of 100 mm and 80 mm, welding flanges of 30 mm, and a thickness of 1.4 mm. The DHBs are subjected to a comprehensive simulation to investigate their axial collision behavior. The Finite Element Analysis (FEA) is conducted over a 60 ms timeframe, using the commercial software Ls-Dyna, version of 6.0.

The FEA material models MAT24 and MAT100 from LS-DYNA’s material library are utilized. MAT24 is also known as MAT_PIECEWISE_LINEAR_PLASTICITY. MAT124 is widely used in engineering fields that require consideration of high-speed deformation, and is still suitable for simulating impact conditions. This study belongs to the impact condition and is different from the quasi-static compression condition. Also, in the team’s research, the effectiveness of using this material was validated by comparing physical experiments with simulation models. On the other hand, MAT100 is a material model specifically designed for solid element spotwelds. The contact within the DHB is defined using the SINGLE-SURFACE contact model; the interaction between the weight and the upper plane is simulated through the SURFACE-TO-SURFACE contact. A 5 mm grid size is chosen for its balance between computational efficiency and accuracy. The time step size for mass scaled solutions is set as −0.00025.

Figure 1c displays the team research of effective stress–strain curves of DP1180 steel at different strain rates, with these material parameters obtained from dynamic tensile tests. During the high-speed tensile test, the required strain rate is obtained by setting the tensile speed of the testing machine, the strain of the specimen in the dynamic tensile process is measured by the DIC system, and the stress characteristics of the specimen are obtained by the testing machine. Finally, the above process is repeated to obtain the engineering stress and strain characteristics at various strain rates. These engineering stress–strain curves are converted into effective stress–strain curves.

### 2.2. Experiments

**Figure 2:**
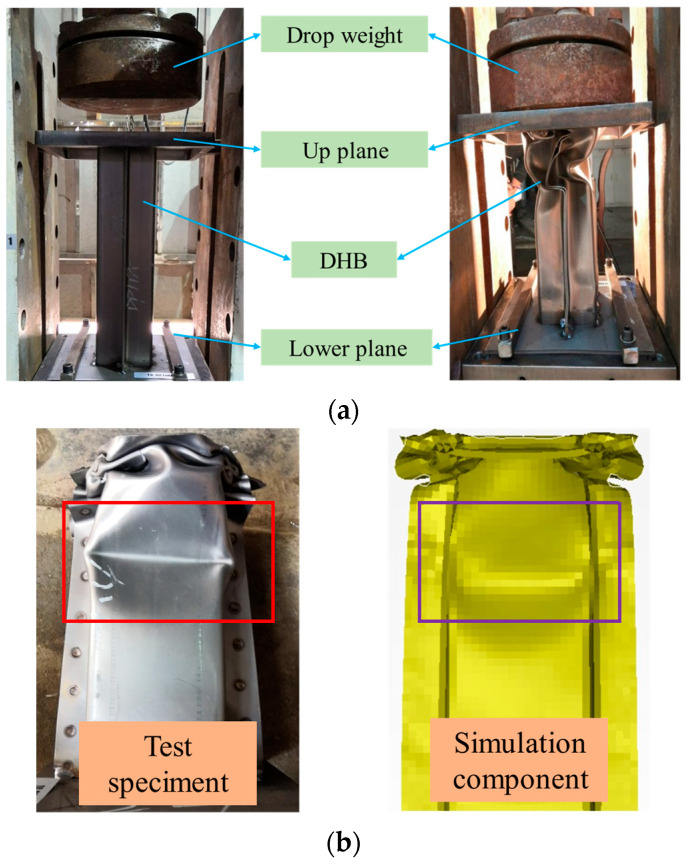
Test and simulation analysis. (**a**) The dynamic drop weight test equipment, and the specimen. (**b**) Comparison of results between test and simulation.

To ascertain the accuracy of the simulation model, impact tests were performed on double-hat beam components, fabricated through stamping techniques and tested at room temperature. Figure 2a depicts the setup for these experiments.

During the tests, a 220 kg drop weight was employed, free-falling from a height of 10 m to impact the upper plane of the DHB. The assembly processes for both the components and the models were kept consistent to ensure comparability.

The constraints applied to the weight and the DHB in the experiment mirrored those in the simulation, ensuring a direct comparison of conditions. Figure 2b presents a comparison of the deformation patterns observed in the experiment with those predicted by the simulation. This comparison reveals a striking similarity in the folded shapes of the DHB, with the bending positions and post-test dimensions of the components aligning almost perfectly. This close resemblance between the test and simulation results underscores the effectiveness and reliability of the simulation model in replicating real-world impact scenarios.

A qualified finite element simulation model should maintain energy balance and accurately capture the proportions and trends of kinetic energy, internal energy, total energy, and hourglass energy. In this research, kinetic energy decreases, internal energy increases, and total energy and hourglass energy are balanced. The energy of the hourglass is less than 5% of the total, and the contact energy is positive.

The evaluation of the DHB impact performance employs a suite of indices on energy absorption and mechanical behavior: Energy Absorption (EA), Specific Energy Absorption (SEA), Mean Crushing Force (MCF), Peak Crushing Force (PCF), Specific Mean Crushing Force (SMCF), and Specific Crushing Force (SCF). Their calculation formulas are shown as follows:

(1) $$EA=\int_{0}^{l}F(x)dx$$

(2) $$SEA=\frac{EA}{SM}$$

(3) $$MCF=\frac{EA}{l}$$

(4) $$SMCF=\frac{MCF}{SM}$$

(5) $$SCF=\frac{MCF}{PCF}$$

where *F*(*x*) is the axial crushing force and *l* denotes the deformation displacement.

**Figure 3:**
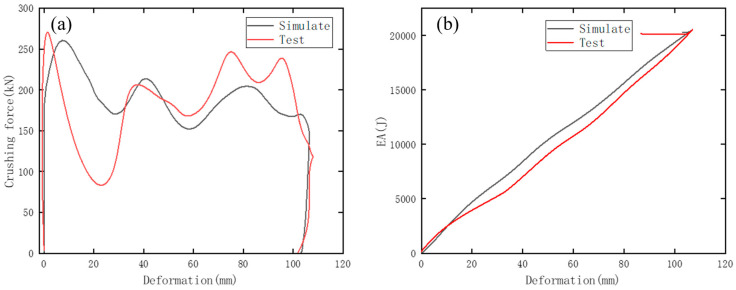
Curve results of test and simulation. Plots of (**a**) crushing force, (**b**) EA.

Figure 3 and Table 1 juxtapose the simulation outcomes with the test data, demonstrating a notable degree of concordance and resemblance.

The simulated Energy Absorption (EA) curve as well as the amplitude and waveform of the crushing force all exhibit a close alignment with their test counterparts. Furthermore, the numerical characteristics derived from the simulation are in consistent agreement with the test observations.

## 3. The Crashworthiness of Modular DHB Structures

In this section, different cell types are proposed and filled into the modular filled structures to improve their crashworthiness.

### 3.1. Three Modular Cell Configurations

**Figure 4:**
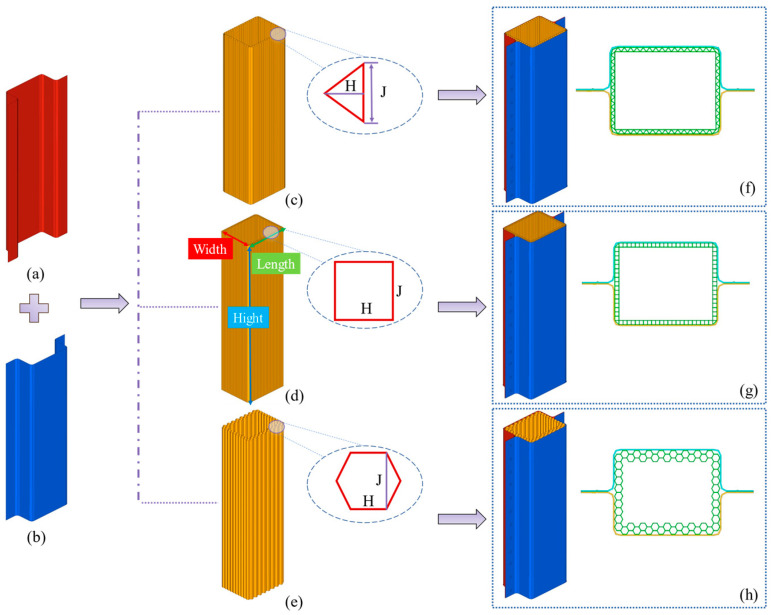
The modular DHBs and their geometric parameters. The (**a**) left and right (**b**) U-shaped plates. The (**c**) triangular, (**d**) quadrangular, and (**e**) hexagonal configurations. The isometric and top plane views (**f**) TR, (**g**) QU, and (**h**) HE.

Figure 4 delineates the geometric parameters of the structures with triangular, quadrilateral, and hexagonal configurations.

These modular structures are characterized by dimensions where the length, width, height, and wall thickness measure 98.49 mm, 78.31 mm, 350 mm, and 0.5 mm; the material is aluminum. The modular DHBs filled with triangular, quadrilateral, and hexagonal Advanced Honeycomb Structures are designated as TR, QU, and HE, respectively. The cell configurations in TR, QU, and HE correspond to isosceles triangles, rectangles, and hexagons. The height and base length of the triangles, as well as the length and width of the rectangles and hexagons, are denoted as H and J, respectively. Table 2 enumerates the specific geometric parameters for the TR, QU, and HE models.

The Dual-layer Hollow Beams (DHBs) and Adaptive Honeycomb configurations possess thicknesses of 1.0 mm and 0.5 mm, respectively. For comparative purposes with the original model, a DHB model named DHB-1.0, mirroring the 1.0 mm thickness, was established. The material properties of Al-6063 encompass a density of 2.7 × 103 kg/m^3^, Young’s modulus of 68 GPa, and Poisson’s ratio of 0.33.

The modular structures are constituted using fully integrated shell elements with three integration points in the thickness direction. Employing a 2 mm mesh size for the configurations has been extensively verified through simulation analysis, demonstrating high accuracy and efficiency. The interaction between the weight and the upper plane is based on surface-to-surface contact, while the interaction between the DHB, the graded structure, and the upper plane is based on self-contact. Coefficients of dynamic and static friction, set at 0.2, regulate these contacts.

### 3.2. Numerical Results

Numerical results for DHB, TR, QU, and HE are presented in Table 3. The main focus metrics are MCF and SMCF. The MCF value is equal to the EA divided by the deformation. The MCF indicates the energy absorbed per unit of deformation; the larger the MCF, the smaller the deformation, and the higher the structural strength. The SMCF can be used to evaluate the resistance strength of unit structure mass. It is feasible to evaluate the crashworthiness of double-hat beams with the metrics of MCF and SMCF.

With the deformation values, it is observed that the descending order is DHB, HE, QU, and TR. Conversely, the Mean Crushing Force (MCF) values exhibit an inverse relationship to the deformation values. The Specific Mean Crushing Force (SMCF) values, in descending order, are HE, TR, QU and DHB, respectively.

Consequently, the MCF and SMCF values of TR, QU, and HE surpass those of DHB, suggesting superior collision resistance for TR, QU, and HE compared to DHB. The Specific Crash Force (SCF) values of TR and QU are greater than those of DHB, while the value for HE is comparable to that of DHB. These results indicate that the inclusion of modular filled structures effectively enhances the crashworthiness and impact stability of DHB.

**Figure 5:**
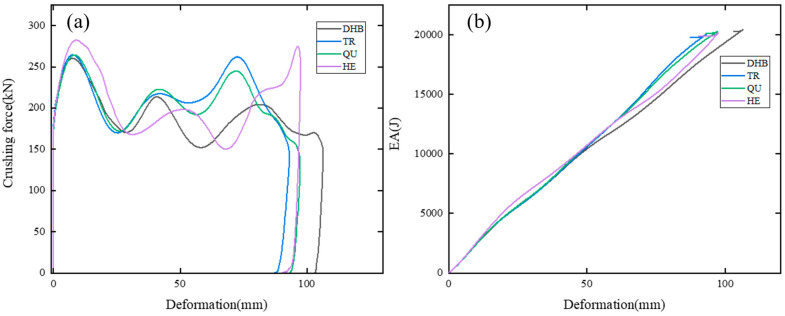
Curve results of DHB, TR, QU, and HE. Plots of (**a**) crushing force, (**b**) EA.

In Figure 5, the crushing force and energy absorption (EA) curves of DHB, TR, QU, and HE are depicted. Figure 5a reveals that the crushing forces and deformations of HE, QU, and TR are higher and smaller, respectively, than those of DHB. In Figure 5b, the EA curves of TR, QU, and HE approximate straight lines, indicating stable crushing forces for these models.

**Figure 6:**
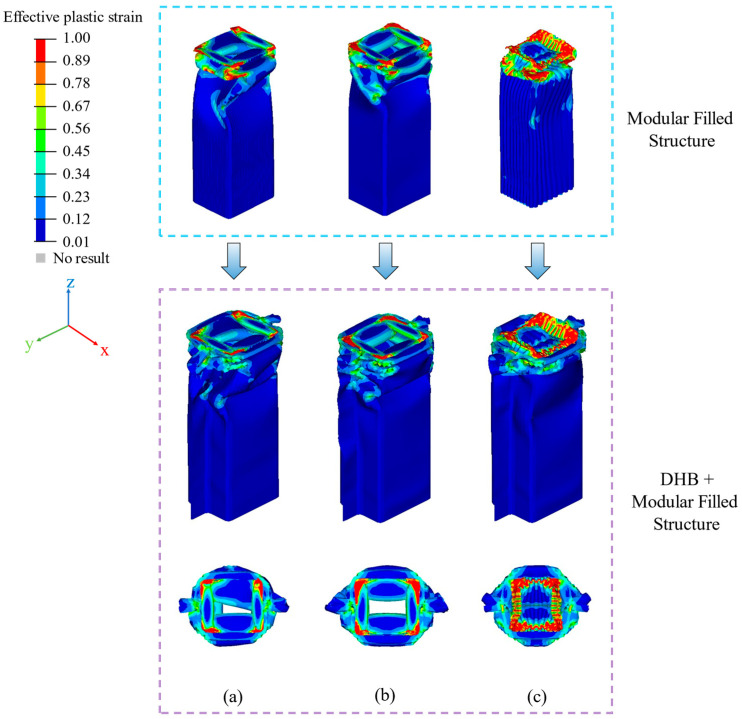
The strain contours of modular filled structures after axial impact. The strain views of (**a**) TR, (**b**) QU, and (**c**) HE.

Figure 6 illustrates the strain contours for various structures, namely DHB, DHB-1.0, TR, QU, and HE. Following axial impact, DHB-1.0 experiences crushing at the upper and lower ends of its U-shaped plates, coupled with bending deformation in the middle and lower regions, significantly diminishing its axial stiffness. In contrast, the deformation of DHB is predominantly concentrated at the top, with localized bending at the lower end.

Notably, TR, QU, and HE exhibit deformation primarily focused on the top of the DHB, showcasing a progressive collapse deformation mode. Consequently, it can be asserted that the deformation modes of TR, QU, and HE exhibit greater stability when compared to DHB and DHB-1.0. Furthermore, from Figure 6, it is evident that the most substantial strains in the modular structures of TR and QU are concentrated in the four corners, while the HE models have noticeable strain concentration along its four edges. The heightened strain in HE can be attributed to two factors: (1) the hexagonal cell size is larger compared to TR and QU, and (2) the cells with two sides adopt a staggered arrangement, resulting in additional corners from cell to cell. Numerical results also confirm that TR, QU, and HE outperform DHB-1.0 and DHB in terms of energy absorption and impact resistance.

## 4. The Effect of Cell Dimensions

The effects of cell dimensions of triangular, quadrangular, and hexagonal shapes on the crashworthiness of DHBs are studied. The load conditions are consistent with Section 2 and Section 3.

### 4.1. The Geometry of Modular Structures

**Figure 7:**
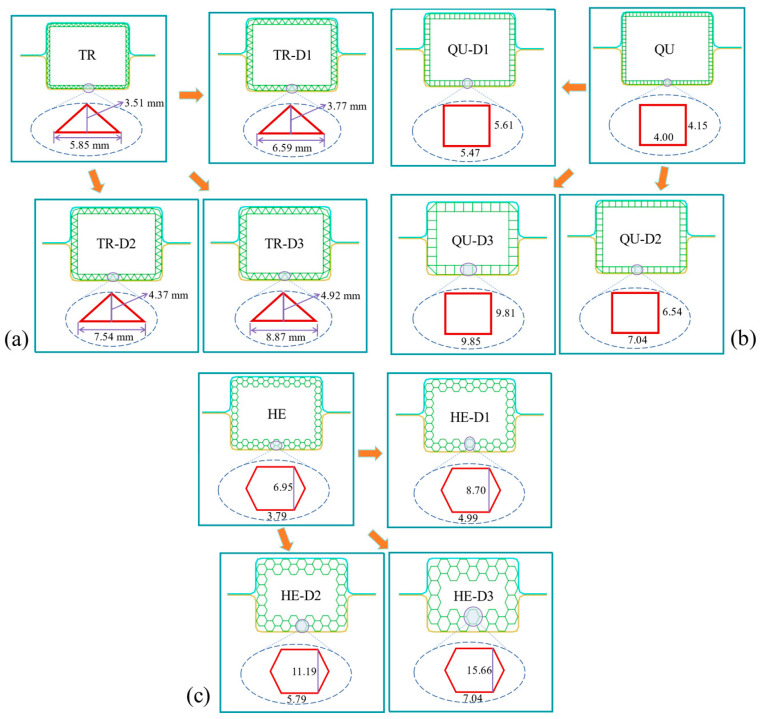
The geometry of these triangular, rectangular, and hexagonal structures with different dimensions. The top plane views (**a**) TR, (**b**) QU, and (**c**) HE models.

Figure 7 and Table 4 show the geometric parameter values of modular triangular, quadrangular, and hexagonal structures. For the triangular structures, the values of J and H are set to 6.59 and 3.77 mm, 7.54 and 4.37 mm, and 8.87 and 4.92 mm, respectively, and they are named TR-D1, TR-D2, and TR-D3. Regarding the quadrangular structures, the values of J and H are set to 5.61 and 5.47 mm, 6.54 and 7.04 mm, and 9.81 and 9.85 mm, respectively, and they are named QU-D1, QU-D2, and QU-D3. For the hexagonal structures, the values of J and H are set to 8.70 and 4.99 mm, 11.19 and 5.79 mm, and 15.66 and 7.04 mm, respectively, and they are named HE-D1, HE-D2, and HE-D3.

The size setting of triangles and hexagons is based on the complete and regular filling scheme after lattice composition, which is formed by combining the shapes to form regular parallelograms and regular hexagons. That is mainly to form a relatively regular modular structure.

When determining the size parameters of shapes as triangles, quadrilaterals, and hexagons for cross-sectional design, a key principle is to systematically increase parameter values in one dimension from small to large. This method aims to create a uniform and continuous changing series for the modular structure, enhancing structural integrity and design efficiency.

### 4.2. Results of These Models

**Figure 8:**
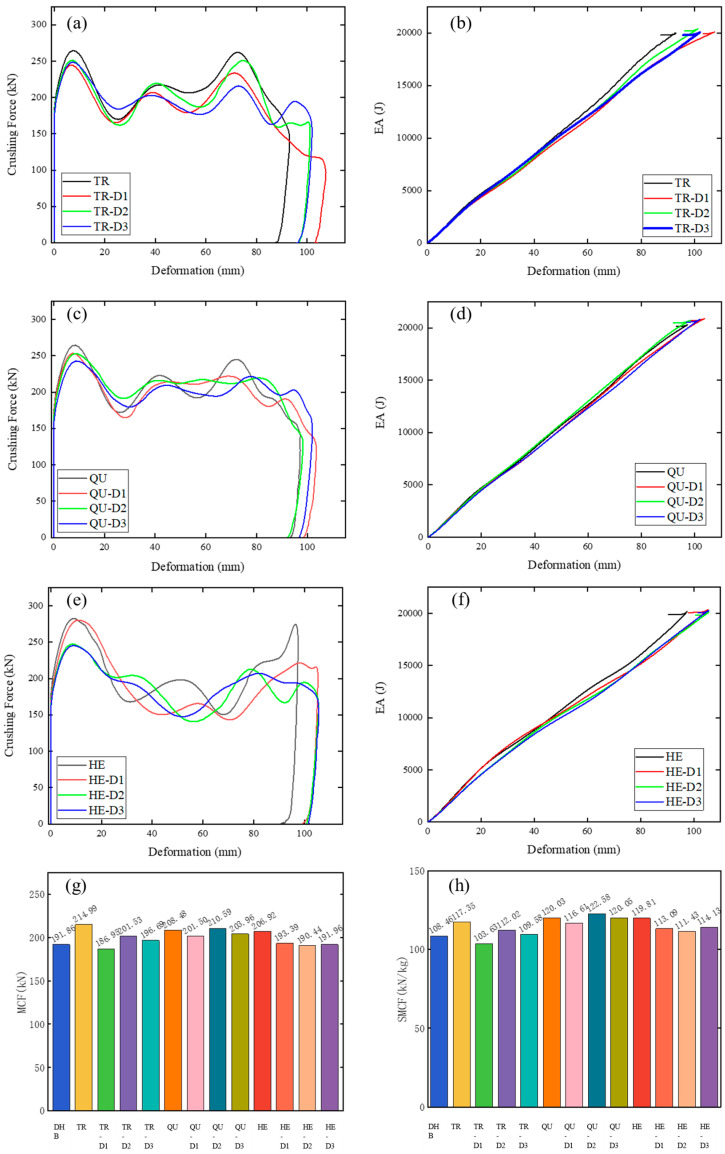
The modular filled structures with different layout characteristics. The (**a**) crushing force and (**b**) EA curves of TR models, plots of (**c**) crushing force and (**d**) EA of QU models, and plots of (**e**) crushing force and (**f**) EA of HE models. Histograms of (**g**) MCF and (**h**) SMCF of these DHB models.

Table 5 and Figure 8 shows the numerical results of TR, TR-D1, TR-D2, TR-D3, QU, QU-D1, QU-D2, QU-D3, HE, HE-D1, HE-D2, and HE-D3. The values of deformation in descending order are TR-D1, HE-D3, HE-D2, HE-D1, QU-D1, QU-D3, TR-D3, TR-D2, QU-D2, HE, QU, and TR. As cell size increases, the values of MCF and SMCF of the HE models decrease first and then increase, and the MCF and SMCF values of the TR and QU models decrease first, then increase, and then decrease. The MCF and SMCF values of the three series are all larger than those of the DHB. The SMCF and MCF values from high to low are QU-D2, QU-D3, QU, HE, TR, QU-D1, HE-D3, HE-D1, TR-D2, HE-D2, TR-D3, and TR-D1. Figure 8 shows the crushing force and EA curves of these models. In Figure 8, the EA value increases linearly with the increase in deformation in the collision process.

**Figure 9:**
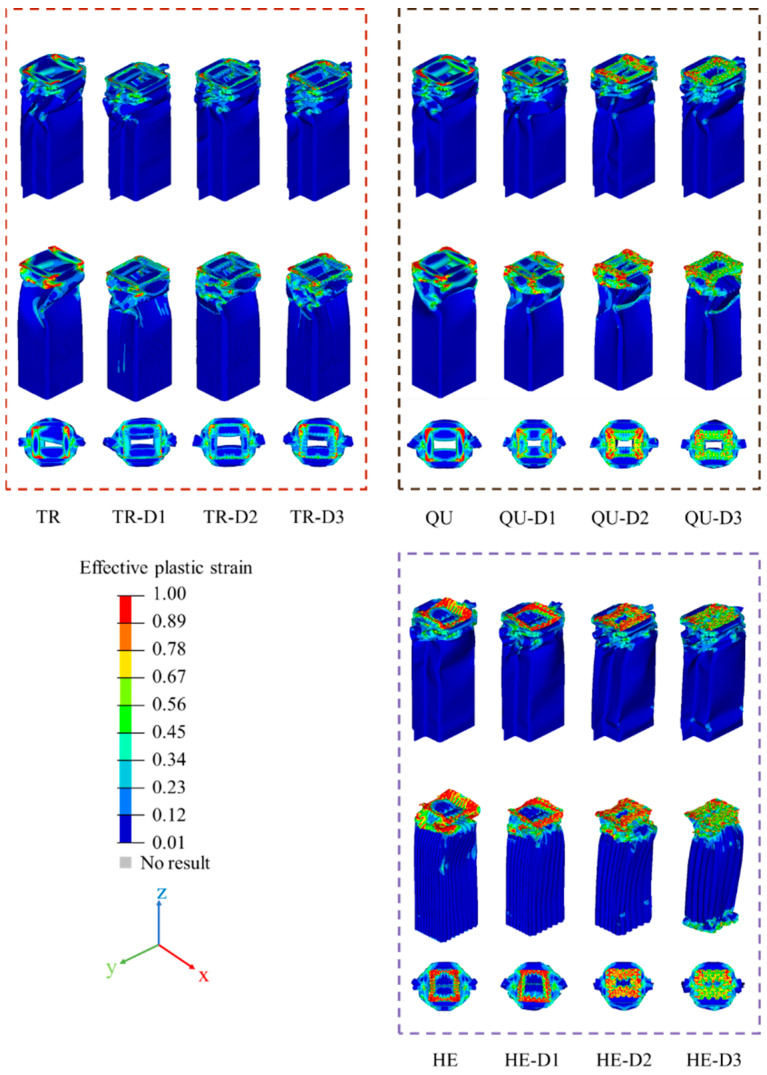
The strain contours of TR-D1, TR-D2, TR-D3, QU-D1, QU-D2, QU-D3, HE-D1, HE-D2, and HE-D3 in isometric and top views.

Figure 9 shows the strain contours of the TR, QU, and HE models.

The deformation of these models is mainly concentrated in their upper parts, which is a progressive crushing deformation mode. TR, TR-D1, TR-D2, TR-D3, QU, QU-D1, QU-D2, QU-D3, HE, HE-D1, HE-D2, and HE-D3 have stably folded deformation at the top of the U-shaped plates. The bottoms of the U-shaped plates of HE-D2 and HE-D3 are slightly crushed. Due to the existence of the modular filled structures, the structural stiffness and crashworthiness of the DHB is significantly improved. It can be noticed that the deformation of HE is larger than that of QU and TR, so the EA of the HE models is larger than that of the other two types of models.

## 5. The Crashworthiness of Modular DHBs

In this section, various modular filled structures under various layout characteristics are proposed to further improve the performance of DHB.

The uses of MCF and SMCF indicators over EA and SEA in experiments and simulations, along with hammer impact on a double-hat beam, are discussed in this section. It explains how the hammer’s kinetic energy transforms during impact and rebound, emphasizing that lower structural strength causes more deformation and lower rebound speeds. This leads to higher EA values for weaker structures. Higher EA and SEA values do not always mean better crashworthiness. The main focus metrics are MCF and SMCF indicators.

### 5.1. The TR with Improved Layout Characteristics

**Figure 10:**
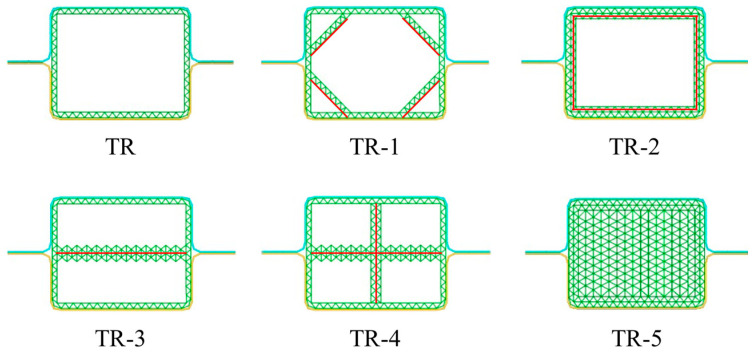
The layout characteristics of five improved triangular DHBs.

The layout characteristics of the triangular structures are improved; these five new structures are proposed and named TR-1, TR-2, TR-3, TR-4, and TR-5, respectively. Figure 10 shows these new TR models.

Among them, TR-1 adds hypotenuses at four corners based on the TR cross-section, TR-2 adds a circle inside the TR cross-section, TR-3 adds a horizontal edge in the middle of the TR cross-section, TR-4 adds a cross in the middle of the TR cross-section, and TR-5 uses triangular cells to fill the whole cross-section of the TR.

**Figure 11:**
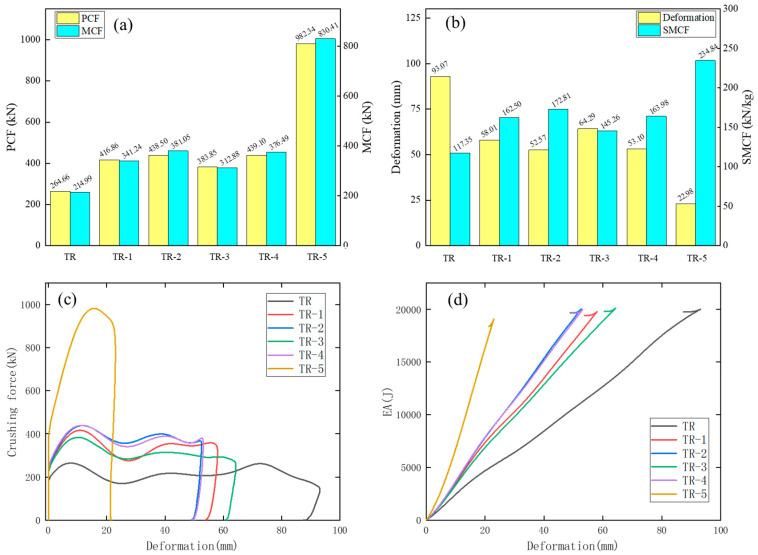
The impact results of TR-A (A = 1, 2, 3, 4, and 5) DHBs. Histograms of (**a**) PCF and MCF as well as (**b**) deformation and SMCF of these TR models. Plots of (**c**) crushing force and (**d**) EA curves.

Table 6 and Figure 11 show the numerical results of TR-1, TR-2, TR-3, TR-4, and TR-5.

The deformation distances of TR-1, TR-2, TR-3, TR-4, and TR-5 are all less than that of TR. These TR models have excellent energy absorption characteristics. The values of deformation in descending order are TR, TR-1, TR-2, TR-3, TR-4, and TR-5, respectively. The values of SM, MCF, and SMCF in descending order are TR-5, TR-4, TR-3, TR-2, TR-1, and TR. Therefore, as the value of SM increases, the values of MCF and SMCF of TR increase. The MCF of TR-2 is higher than that of TR-1, indicating that a circle of triangular microstructures in the inner side of TR has a better effect than directly adding hypersonic edges to the four inner corners of TR. MCF is an important indicator and should be discussed in detail. The MCF of TR-4 is higher than that of TR-3, indicating that adding horizontal and vertical edges to TR has a better effect than directly adding a horizontal edge. The addition of horizontal and vertical edges increases the contact area, and the extrusion pressure between cells increases after impact. The MCF of TR-5 is higher than that of the other TR models, indicating that the effect of fully filling the structure with triangular microstructures is higher than that of the other filling methods. Compared with the TR, the SMCF values of TR-1, TR-2, TR-3, TR-4 and TR-5 are increased by 38.47%, 47.26%, 23.78%, 39.73%, and 100.12%, respectively. It can be concluded that the crashworthiness of the TR model increases with the increases in cell number. The SCF values of these TR models are all larger than 81%; this shows that the crushing forces of these models are very stable. The deformation characteristics are also regular and uniform.

From Figure 11c, the crushing forces of these TR models are very stable. The fluctuation trend and waveform of the crushing force curves of TR-1, TR-2, TR-3, and TR-4 are similar, indicating that the structural stiffness of these four models is very similar and the crashworthiness of the structures is good. The PCF and MCF values of TR-5 reach 982.34 and 830.41 kN, which are much higher than those of the other TR models. Figure 11d depicts the EA curves of these TR models. The EA values of these TR models show a linear increase with the increases in deformation, and TR-5 has the fastest growth rate and the steepest slope among these models. This shows that the crashworthiness of TR-5 is better than that of the other TR models. The triangular AHS filling is more conducive to improving the crashworthiness and stability of the DHB.

**Figure 12:**
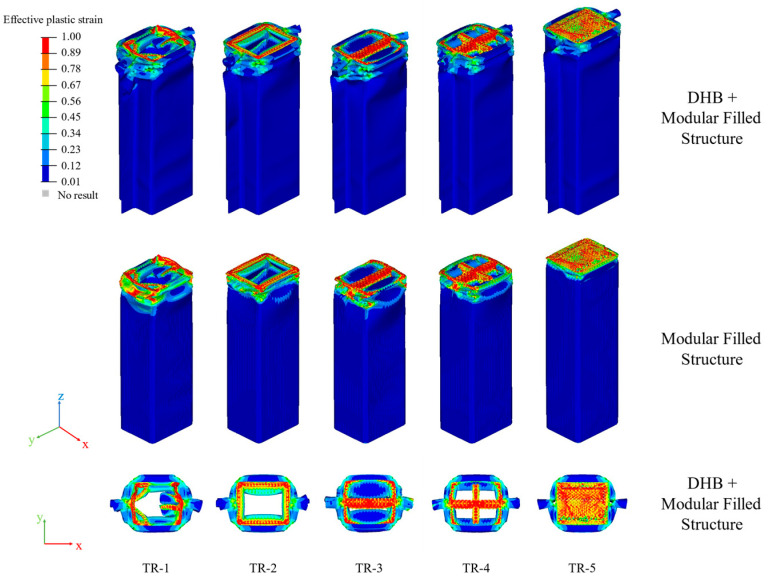
The strain contours of TR-1, TR-2, TR-3, TR-4, and TR-5 in isometric and top plane views.

Figure 12 shows the strain contours of these TR models.

It is found that the axial crashworthiness of these TR models is very stable. The deformation modes of TR-1, TR-2, TR-3, TR-4, and TR-5 are all progressive crushing deformation modes, which are more stable compared to the DHB and TR models. The strain is mainly concentrated on the top of these models, and the strain concentration of TR-1, TR-2, TR-3, TR-4, and TR-5 is more significant than that of TR. With the increase in the number of micro-cells, the phenomenon of strain concentration is more obvious.

### 5.2. The QU with Improved Layout Characteristics

The performance of the QU models is studied, and five improved layout structures, named QU-1, QU-2, QU-3, QU-4, and QU-5, are proposed. Among them, QU-1 adds two diagonal lines inside the rectangle structures based on QU, QU-2 adds small rectangle cells on the microstructures of QU-1, QU-3 adds a horizontal line in the middle of the QU cross-section, QU-4 adds a cross in the middle of the QU cross-section, and QU-5 fills the whole cross-section of QU with the rectangle structures.

**Figure 13:**
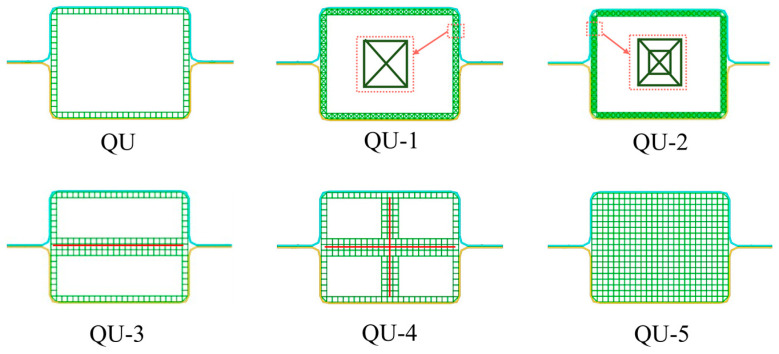
The layout characteristics of five improved quadrangular DHBs.

The numerical results of QU, QU-1, QU-2, QU-3, QU-4, and QU-5 are shown in Table 7 and Figure 13.

**Figure 14:**
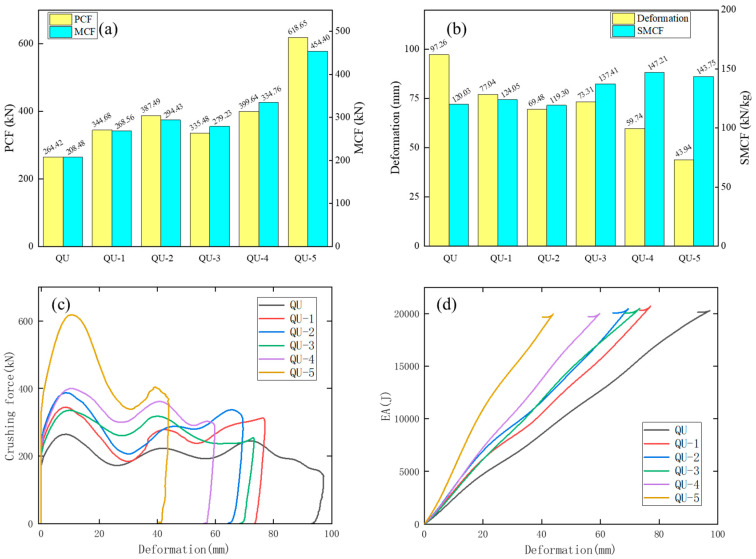
The impact results of QU-A (A = 1, 2, 3, 4, and 5) DHB with different structural layering are investigated. (**a**) Histograms of PCF and MCF of these QU models. (**b**) Histograms of Deformation and SMCF of these QU models. Plots of (**c**) crushing force-deformation and (**d**) EA-deformation curves of these QU models.

The deformations of QU-1, QU-2, QU-3, QU-4, and QU-5 are all smaller than that of QU. The results show that these QU models have great energy absorption and impact resistance properties. The values of MCF in descending order are QU-5, QU-4, QU-2, QU-3, QU-1, and QU, and the values of SMCF in descending order are QU-5, QU-3, QU-1, QU, QU-4, and QU-2. QU-3 and QU-4 have the largest values of SCF, and their impact performance is the most stable among these models. This is because the number, arrangement, and stiffness of the cells of QU-3 and QU-4 are appropriate. The SMCF value of QU-5 is 19.77% higher than that of the QU model. From Figure 14 the crushing forces of these QU models are very stable. The crushing force waveforms of QU-1, QU-2, QU-3, and QU-4 are similar, indicating that their structural stiffness is similar. The PCF of QU-5 reaches 618.65 kN which is much higher than that of the other QU models. From Figure 14d, the EA values of these QU models show a linear increase with the increase in deformation, which shows that the crushing force and energy absorption characteristics of these models are very stable. QU-5 has the fastest growth rate and the highest slope.

**Figure 15:**
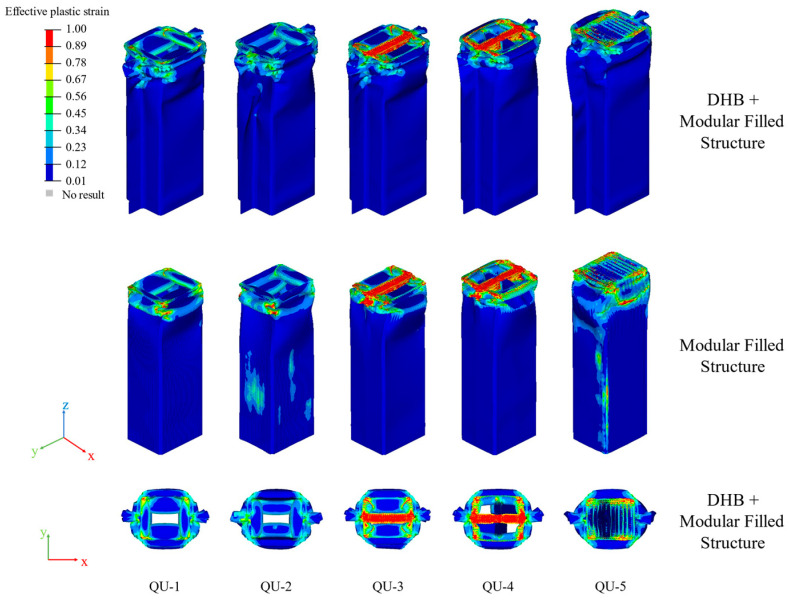
The strain contours of QU-1, QU-2, QU-3, QU-4, and QU-5 in isometric and top plane views.

Figure 15 shows the deformation patterns of these QU models. The deformation of these QU models is mainly concentrated in their upper parts, and their lower parts remain stable after impact.

The deformation modes of QU-1, QU-2, QU-3, QU-4, and QU-5 are all progressive axial collapse. The AHSs of these QU models have greater deformation compared with the TR models.

### 5.3. The HE with Improved Layout Characteristics

**Figure 16:**
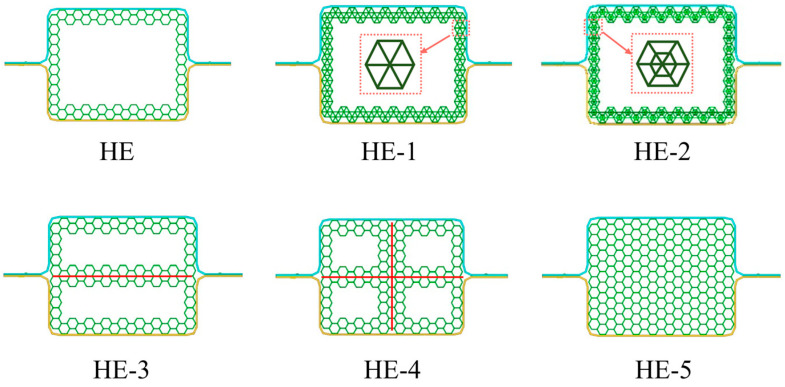
The layout characteristics of five improved hexagonal DHBs.

The axial impact performance of HE with improved AHSs is studied. Five improved layout structures, named HE-1, HE-2, HE-3, HE-4, and HE-5, are proposed, as shown in Figure 16. HE-1 adds three diagonal lines inside the hexagonal cells based on HE, HE-2 adds small hexagonal structures inside the cells of HE-1, HE-3 adds a horizontal line in the middle of the HE cross-section, HE-4 adds a cross in the middle of the HE cross-section, and HE-5 fills the whole cross-section of HE with hexagonal structures.

**Figure 17:**
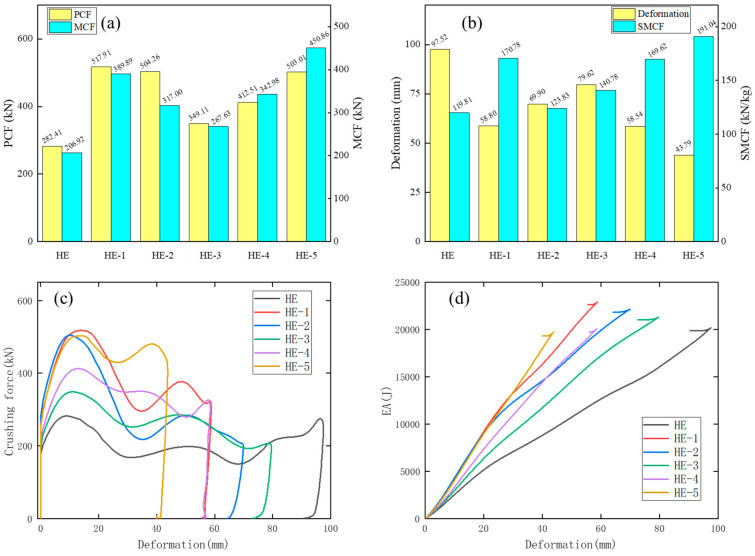
The impact results of HE-A (A = 1, 2, 3, 4, and 5) models. Histograms of (**a**) PCF and MCF, (**b**) deformation and SMCF of these HE models. Plots of (**c**) crushing force–deformation and (**d**) EA–deformation curves of these HE models.

The numerical results of HE, HE-1, HE-2, HE-3, HE-4, and HE-5 are shown in Table 8 and Figure 17.

The deformation distances of HE-1, HE-2, HE-3, HE-4, and HE-5 are all smaller than those of HE, and this implies that these HE models have great energy absorption characteristics. The deformation values are HE, HE-3, HE-2, HE-1, HE-4, and HE-5 in descending order. The values of MCF and SMCF in descending order are HE-5, HE-1, HE-4, HE-2, HE-3, and HE. After axial compression, it is found that the SMCF value of HE-3 is significantly smaller than that of HE-4 and HE-5, indicating that more cells are conducive to improving the crashworthiness of the structure. The crash-resistant performance of HE-1 is significantly higher than that of HE-2 when three diagonal lines are added to the hexagonal microstructure. The main reason is that the stiffness of the cells of HE-1 is less than that of HE-2, and the wavelength of its folds during crushing is less than that of HE-2. HE-5 and HE-4 obtain the maximum SCF values. From Figure 17c, the waveforms of the crushing force curves of HE-3, HE-4, and HE-5 collision are similar, and their compression forces are stable. The crushing force curves of HE-1 and HE-2 are similar. Their initial crushing forces are very large, but later they significantly decrease. From Figure 17d, the EA values of these HE models increase rapidly with the increase in deformation. But HE-5 has the fastest growth rate and the highest slope.

**Figure 18:**
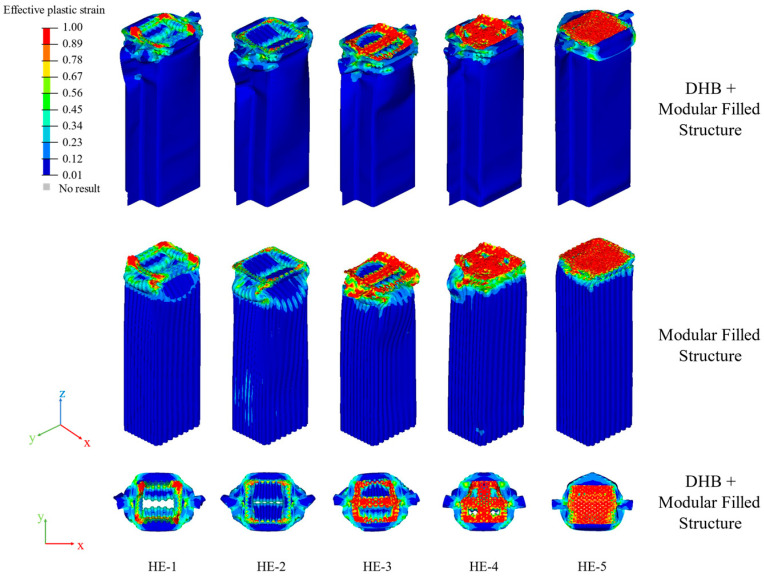
The strain contours of HE-1, HE-2, HE-3, HE-4, and HE-5 in isometric and top plane views.

Figure 18 shows the deformation patterns of these HE models. From the strain contours, the deformation modes of the HE models are stable.

The deformation modes of HE-1, HE-2, HE-3, HE-4, and HE-5 are all progressive collapse. For the deformation of modular filled structures, HE-1 and HE-2 show inner convex deformation and they have larger wavelengths of folds compared with the other models. This is the reason why their initial crushing force is large and in the later period drops obviously. For HE-3, the inner structure is drum-shaped outward. For HE-4 and HE 5, the lower parts remain stable, and the top of the structures suffer severe extrusion deformation.

### 5.4. Comparative Discussion among Modular Structures

**Figure 19:**
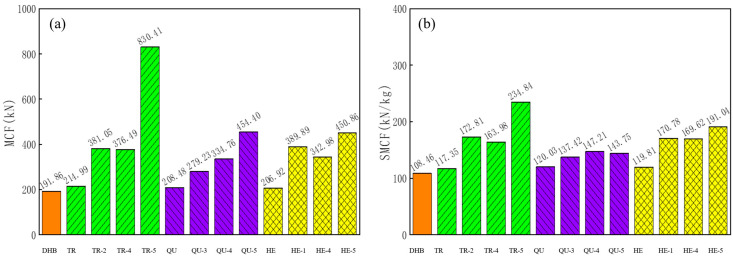
Histograms of (**a**) MCF and (**b**) SMCF of these DHB, TR, QU, and HE models.

In this subsection, three models with the maximum SMCF values are selected from each type of modular model. Figure 19 and Table 9 show the results of these models and the results of DHB, and we have selected three base models and two modular structures with better performance for comparative analysis with the original simulated DHB model.

The simulation results of the key crashworthiness indices of the structure are presented in Table 9, and, as described, the other nine modular structures of the DHB have greater improvements in deformation, MCF, and SMCF compared to those of DHB. In particular, the modular filled structure shows a significant improvement in MCF and SMCF values. For example, regarding the MCF value, the MCF values of TR-5 and HE-5 showed the most significant improvement, reaching 332.8% and 136.8%, respectively. Regarding the SMCF value, except for TR-5, the HE models showed a significant improvement in SMCF value. Taking HE-1 and HE-5 as examples, they increased by 57.5% and 76.1%, respectively. The DHBs with infill obtain a lot of improvement in bending stiffness, and there is an increase in mass, but it is relatively small compared to the total mass. The DHBs with infill can significantly increase the EA capacity and stability. This proves once again that the modular filled DHBs can significantly increase not only the crashworthiness but also the stability of the structure.

### 5.5. Manufacturability of These Structures

In this study, while the results have yet to be experimentally validated, the importance of validation is clarified. In subsequent studies, a 3D printing method will be utilized for creating samples for physical experiments and verification analysis, with numerous capable factories available for production. Comparative studies between simulation and experimentation will be conducted.

Future research will explore various manufacturing methods, including aluminum extrusion. This widely used metal processing technique finds application across diverse industries and fields. Aluminum extruded products are pivotal in transportation, including automotive components, train body structures, and aerospace components, owing to their high strength, lightweight properties, excellent formability, and corrosion resistance. These attributes contribute significantly to reducing the overall weight of transportation vehicles. The structure of this study lends itself well to practical manufacturing.

## 6. Discussion

This research aims to innovate traditional beam design by introducing unique cross-section shapes that enhance energy absorption and deformation behavior. The innovations of this study lie in the modular methods by arranging and filling hexagonal, quadrilateral, and triangular aluminum graded structures in DHB and explore their influencing mechanism. Simultaneously, introducing a combination of different materials, namely the modular fusion of modular aluminum alloy graded structures with high-strength steel, a new modular DHB structure was constructed with the aim of reducing structural mass (SM) and enhancing axial energy absorption characteristics. Three types of modular filled structures are innovatively proposed and filled into the DHBs, the energy absorption effects and deformation patterns of DHB were systematically studied, and a parameter analysis of the influence of modular aluminum graded structures on DHB impact performance was conducted.

## 7. Conclusions

Firstly, the research clarifies the motivation and importance of the study. The necessity of investigating modular structures is demonstrated by the current research status of DHB structures and modular structures. Then, specific and clear research objectives have been established, namely, to utilize modular filling methods to optimize the filling of the cross-section of the double-cap beam, thereby enhancing the mechanical properties of the DHB structure. This section summarizes the main results obtained from the study, encompassing both qualitative and quantitative findings. Additionally, the significance and prospects of the results are elaborated upon, looking forward to future research directions and potential areas for improvement. The research trend of modular structures is explored. Some meaningful conclusions are drawn.

1. The dimension of the modular structure also has a significant change in the overall collision performance of the DHB structure. The SMCF of the three basic models is improved, among which TR-D2, QU-D2, and HE-D3 are significantly improved by 3.18%, 13.02%, and 5.23%, respectively.
2. Compared with the original model, the SMCF of the improved modular DHB structure has been improved greatly, among which TR-5, QU-5, and HE-5 have the best performance, which has increased by 116.53%, 32.54%, and 78.9%, respectively.
3. In the optimization process of modular filled structures, the axial crushing of the triangle element is better than that of the quadrilateral element and hexagon element.
4. Appropriately adding units contributes to enhancing the structural durability. In the triangular improved modular model, reinforcing modularization at the edges enhances the mean crushing force. In the quadrangular improved modular model, the cross-shaped modular filling approach proves more conducive to enhancing energy absorption effects.
5. In conclusion, compared with the traditional hollow DHBs, by innovatively proposing modular filled structures inside DHBs, the composite structures exhibit unique energy absorption characteristics and impact resistance characteristics, and it is practicable to select and match various filling methods based on their performance. Furthermore, future research will explore the cross arrangement of different modular unit cells to achieve superior energy absorption and crush characteristics. This study reveals the key characteristics of axial vehicle impact and addresses the limitations of previous research. It holds significant reference value for automotive engineers and scholars looking to delve deeper into the axial crash safety, platform modularization, and lightweight design of electric vehicles.

## Figures and Tables

**Table 1:** Numerical results of simulation and three tests.

	MCF (kN)	SEA (J/kg)	SMCF (kN/kg)	Def. (mm)	PCF (kN)	SM (kg)	EA (J)
Simulation	191.86	11,543.98	108.46	106.44	260.52	1.769	20,421.3
Test	191.97	11,612.9	108.52	107.01	268.80	1.769	20,543.3

**Table 2:** Geometric parameter values of TR, QU and HE.

Models	J (mm)	H (mm)	N	M	Length (mm)	Width (mm)	Height (mm)
TR	5.85	3.51	31	23	98.49	78.31	350
QU	4.15	4.00	23	17	98.49	78.31	350
HE	6.95	3.79	15	10	98.49	78.31	350

Note: N and M represent the number of cells in the length and width directions, respectively.

**Table 3:** Numerical results of these DHB models.

	MCF (kN)	SEA (J/kg)	SMCF (kN/kg)	SCF (%)	SM (kg)	PCF (kN)	Def. (mm)	EA (J)
TR	214.99	10,922.2	117.35	81.23	1.832	264.66	93.07	20,009.4
QU	208.48	11,673.2	120.03	78.84	1.737	264.42	97.26	20,276.3
HE	206.92	11,684.6	119.81	73.27	1.730	282.41	97.52	20,179.3
DHB	191.86	11,543.98	108.46	73.64	1.769	260.52	106.44	20,421.3

**Table 4:** The geometric parameter values of these structures.

Model	J (mm)	H (mm)	N	M	Length (mm)	Width (mm)	Height (mm)
TR	5.85	3.51	31	23	98.49	78.31	350
TR-D1	6.59	3.77	27	19	98.49	78.31	350
TR-D2	7.54	4.37	23	17	98.49	78.31	350
TR-D3	8.88	4.92	19	15	98.49	78.31	350
QU	4.15	4.00	23	17	98.49	78.31	350
QU-D1	5.61	5.47	16	12	98.49	78.31	350
QU-D2	6.54	7.04	12	10	98.49	78.31	350
QU-D3	9.81	9.85	8	6	98.49	78.31	350
HE	6.95	3.79	15	10	98.49	78.31	350
HE-D1	8.70	4.99	11	8	98.49	78.31	350
HE-D2	11.19	5.79	9	6	98.49	78.31	350
HE-D3	15.66	7.04	7	4	98.49	78.31	350

**Table 5:** Numerical results of these models.

Model	SM (kg)	PCF (kN)	Def. (mm)	EA (J)	MCF (kN)	SEA (J/kg)	SMCF (kN/kg)	SCF (%)
TR	1.832	264.66	93.07	20,009.4	214.99	10,922.2	117.35	81.23
TR-D1	1.804	244.57	107.50	20,096.8	186.95	11,140.13	103.63	76.44
TR-D2	1.799	251.20	101.24	20,403.3	201.53	11,341.47	112.02	80.23
TR-D3	1.795	248.82	101.96	20,054.9	196.69	11,172.66	109.58	79.05
QU	1.737	264.42	97.26	20,276.3	208.48	11,673.2	120.03	78.84
QU-D1	1.728	253.30	103.61	20,877.4	201.50	12,081.83	116.61	79.55
QU-D2	1.718	253.04	98.32	20,704.1	210.59	12,051.28	122.58	83.22
QU-D3	1.699	242.65	101.98	20,799.7	203.96	12,242.32	120.05	84.06
HE	1.727	282.41	97.52	20,179.3	206.92	11,684.60	119.81	73.27
HE-D1	1.710	280.14	105.31	20,365.5	193.39	11,909.65	113.09	69.03
HE-D2	1.709	247.47	105.57	20,104.8	190.44	11,764.07	111.43	76.95
HE-D3	1.682	245.38	105.61	20,273.4	191.96	12,053.15	114.13	78.23

**Table 6:** Numerical results for the TR models.

	SM (kg)	PCF (kN)	Def. (mm)	EA (J)	MCF (kN)	SEA (J/kg)	SMCF (kN/kg)	SCF (%)
TR	1.832	264.66	93.07	20,009.4	214.99	10,922.2	117.35	81.23
TR-1	2.100	416.86	58.01	19,795.5	341.24	9426.4	162.50	81.86
TR-2	2.205	438.50	52.57	20,031.8	381.05	9084.7	172.81	86.90
TR-3	2.154	383.85	64.29	20,114.5	312.88	9338.2	145.26	81.51
TR-4	2.296	439.10	53.10	19,990.0	376.49	8667.7	163.98	85.74
TR-5	3.536	982.34	22.98	19,083.7	830.41	5397.0	234.84	84.53

**Table 7:** Numerical results for the QU models.

	SM (kg)	PCF (kN)	Def. (mm)	EA (J)	MCF (kN)	SEA (J/kg)	SMCF (kN/kg)	SCF (%)
QU	1.737	264.42	97.26	20,276.3	208.48	11,673.20	120.03	78.84
QU-1	2.165	344.68	77.04	20,690.5	268.57	9556.81	124.05	77.92
QU-2	2.468	387.49	69.48	20,456.2	294.43	8288.57	119.30	75.98
QU-3	2.032	335.48	73.31	20,469.8	279.23	10,073.72	137.42	83.23
QU-4	2.274	399.64	59.74	19,999.3	334.76	8794.77	147.21	83.77
QU-5	3.161	618.65	43.94	19,968.2	454.40	6317.05	143.75	73.45

**Table 8:** Numerical results for the HE models.

	SM (kg)	PCF (kN)	Def. (mm)	EA (J)	MCF (kN)	SEA (J/kg)	SMCF (kN/kg)	SCF (%)
HE	1.727	282.41	97.52	20,179.3	206.92	11,684.60	119.81	73.27
HE-1	2.283	517.91	58.80	22,924.4	389.89	10,041.35	170.78	75.28
HE-2	2.560	504.26	69.90	22,156.4	317.00	8654.84	123.83	62.86
HE-3	1.901	349.11	79.62	21,308.1	267.63	11,208.89	140.78	76.66
HE-4	2.022	412.51	58.54	20,079.2	342.98	9930.37	169.62	83.14
HE-5	2.36	503.01	43.79	19,742.0	450.86	8365.25	191.04	89.63

**Table 9:** Numeric results of key parameters.

		SM (kg)	PCF (kN)	Def. (mm)	EA (J)	MCF (kN)	SEA (J/kg)	SMCF (kN/kg)	SCF (%)
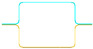	DHB	1.769	260.52	106.44	20,421.3	191.86	11,543.98	108.46	73.65
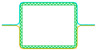	TR	1.832	264.66	93.07	20,009.4	214.99	10,922.20	117.35	81.23
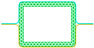	TR-2	2.205	438.50	52.57	20,031.8	381.05	90,84.70	172.81	86.90
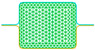	TR-5	3.536	982.34	22.98	19,083.7	830.41	5397.00	234.85	84.53
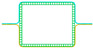	QU	1.737	264.42	97.26	20,276.3	208.48	11,673.20	120.03	78.84
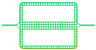	QU-3	2.032	335.48	73.31	20,469.8	279.23	10,073.7	137.42	83.23
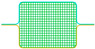	QU-5	3.161	618.65	43.94	19,968.2	454.40	6317.05	143.75	73.45
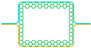	HE	1.730	282.41	97.52	20,179.3	206.92	11,684.60	119.81	73.27
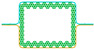	HE-1	2.283	517.91	58.797	22,924.4	389.89	10,041.40	170.78	75.28
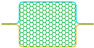	HE-5	2.36	503.01	43.787	19,742.0	450.86	8365.25	191.04	89.63

## Data Availability

The raw data supporting the conclusions of this article will be made available by the authors on request.

## Abbreviations

EA	Energy absorption
SEA	Specific energy absorption
MCF	Mean crushing force
SMCF	Specific mean crushing force
SCF	Specific crushing force
PCF	Peak crushing force
DHB	Double-hat beam
TR	Triangular filled structure
QU	Quadrilateral filled structure
HE	Hexagonal filled structure
TR-D1	Triangular filled structure dimension 1
TR-D2	Triangular filled structure dimension 2
TR-D3	Triangular filled structure dimension 3
QU-D1	Quadrilateral filled structure dimension 1
QU-D2	Quadrilateral filled structure dimension 2
QU-D3	Quadrilateral filled structure dimension 3
HE-D1	Hexagonal filled structure dimension 1
HE-D2	Hexagonal filled structure dimension 2
HE-D3	Hexagonal filled structure dimension 3
TR-1	Triangular filled structure section 1
TR-2	Triangular filled structure section 2
TR-3	Triangular filled structure section 3
TR-4	Triangular filled structure section 4
TR-5	Triangular filled structure section 5
QU-1	Quadrilateral filled structure section 1
QU-2	Quadrilateral filled structure section 2
QU-3	Quadrilateral filled structure section 3
QU-4	Quadrilateral filled structure section 4
QU-5	Quadrilateral filled structure section 5
HE-1	Hexagonal filled structure section 1
HE-2	Hexagonal filled structure section 2
HE-3	Hexagonal filled structure section 3
HE-4	Hexagonal filled structure section 4
HE-5	Hexagonal filled structure section 5

## References

[1] Ding S., Sun M., Li Y., Ma W., Zhang Z. (2022). Novel deployable panel structure integrated with thick origami and morphing bistable composite structures. Materials.

[2] Wen Z., Li M. (2021). Compressive properties of functionally graded bionic bamboo lattice structures fabricated by fdm. Materials.

[3] Bi G., Yin J., Wang Z., Jia Z. (2020). Micro fracture behavior of composite honeycomb sandwich structure. Materials.

[4] Sakaridis E., Karathanasopoulos N., Mohr D. (2022). Machine-learning based prediction of crash response of tubular structures. Int. J. Impact Eng..

[5] Li Q.Q., Li E., Chen T., Wu L., Wang G.Q., He Z.C. (2021). Improve the frontal crashworthiness of vehicle through the design of front rail. Thin-Walled Struct..

[6] Li Z., Wang Z., Wang X., Zhou W. (2020). Bending behavior of sandwich beam with tailored hierarchical honeycomb cores. Thin-Walled Struct..

[7] Xu M., Zhao Z., Wang P., Duan S., Lei H., Fang D. (2022). Mechanical performance of bio-inspired hierarchical honeycomb metamaterials. Int. J. Solids Struct..

[8] Lu B., Shen C., Zhang J., Zheng D., Zhang T. (2021). Study on energy absorption performance of variable thickness CFRP/aluminum hybrid square tubes under axial loading. Compos. Struct..

[9] Herakovich C.T., Aboudi J., Lee S.W., Strauss E.A. (1988). Damage in composite laminates: Effects of transverse cracks. Mech. Mater..

[10] Tsang H.H., Tse K.M., Chan K.Y., Lu G., Lau A.K. (2019). Energy absorption of muscle-inspired hierarchical structure: Experimental investigation. Compos. Struct..

[11] Fan H., Luo Y., Yang F., Li W. (2018). Approaching perfect energy absorption through structural hierarchy. Int. J. Eng. Sci..

[12] Xu X., Zhang Y., Wang J., Jiang F., Wang C.H. (2018). Crashworthiness design of novel hierarchical hexagonal columns. Compos. Struct..

[13] Li W., Luo Y., Li M., Sun F., Fan H. (2018). A more weight-efficient hierarchical hexagonal multi-cell tubular absorber. Int. J. Mech. Sci..

[14] Liu S., Tong Z., Tang Z., Liu Y., Zhang Z. (2015). Bionic design modification of non-convex multi-corner thin-walled columns for improving energy absorption through adding bulkheads. Thin-Walled Struct..

[15] Tao Y., Li W., Wei K., Duan S., Wen W., Chen L., Fang D. (2019). Mechanical properties and energy absorption of 3D printed square hierarchical honeycombs under in-plane axial compression. Compos. Part B Eng..

[16] Wang Z., Li Z., Shi C., Zhou W. (2019). Mechanical performance of vertex-based hierarchical vs square thin-walled multi-cell structure. Thin-Walled Struct..

[17] Kim C.H., Mijar A.R., Arora J.S. (2001). Development of simplified models for design and optimization of automotive structures for crashworthiness. Struct. Multidiscip. Optim..

[18] Li Q., Wu L., Hu L., Li E., Zhong Y., Song K. (2022). Bionic polycellular structures for axial compression. Int. J. Mech. Sci..

[19] Li Q., Wu L., Hu L., Li E., Zou T., Liu X. (2023). Parametric analysis on axial compression performance of bio-inspired porous lattice structures. Thin-Walled Struct..

[20] Mayer R.R., Lin C.H., Wang J.T. Math-Based Performance Evaluation of an Experimental Car: Frontal Impact Crashworthiness. Proceedings of the International Design Engineering Technical Conferences and Computers and Information in Engineering Conference.

[21] Duan L., Xiao N., Hu Z., Li G., Cheng A. (2017). An efficient lightweight design strategy for body-in-white based on implicit parameterization technique. Struct. Multidiscip. Optim..

[22] Sun G., Wang X., Fang J., Pang T., Li Q. (2021). Parallelized optimization design of bumper systems under multiple low-speed impact loads. Thin-Walled Struct..

[23] Sun G., Pang T., Fang J., Li G., Li Q. (2017). Parameterization of criss-cross configurations for multiobjective crashworthiness optimization. Int. J. Mech. Sci..

[24] Wang Z., Zhang J., Li Z., Shi C. (2020). On the crashworthiness of bio-inspired hexagonal prismatic tubes under axial compression. Int. J. Mech. Sci..

[25] Sun G., Xu F., Li G., Li Q. (2014). Crashing analysis and multiobjective optimization for thin-walled structures with functionally graded thickness. Int. J. Impact Eng..

[26] Li Y., Lin Z., Jiang A., Chen G. (2003). Use of high strength steel sheet for lightweight and crashworthy car body. Mater. Des..

[27] Zhang Y., Lai X., Zhu P., Wang W. (2006). Lightweight design of automobile component using high strength steel based on dent resistance. Mater. Des..

[28] Deb A., Mahendrakumar M.S., Chavan C., Karve J., Blankenburg D., Storen S. (2004). Design of an aluminium-based vehicle platform for front impact safety. Int. J. Impact Eng..

[29] Cui X., Zhang H., Wang S., Zhang L., Ko J. (2011). Design of lightweight multi-material automotive bodies using new material performance indices of thin-walled beams for the material selection with crashworthiness consideration. Mater. Des..

[30] Liu Q., Lin Y., Zong Z., Sun G., Li Q. (2013). Lightweight design of carbon twill weave fabric composite body structure for electric vehicle. Compos. Struct..

[31] Duan S., Tao Y., Han X., Yang X., Hou S., Hu Z. (2014). Investigation on structure optimization of crashworthiness of fiber reinforced polymers materials. Compos. Part B Eng..

[32] Hesse S.H., Lukaszewicz D.J., Duddeck F. (2015). A method to reduce design complexity of automotive composite structures with respect to crashworthiness. Compos. Struct..

[33] Li Q., Wu L., Hu L., Miao X., Liu X., Zou T. (2023). A sinusoidal beam lattice structure with negative Poisson’s ratio property. Aerosp. Sci. Technol..

[34] Nia A.A., Parsapour M. (2014). Comparative analysis of energy absorption capacity of simple and multi-cell thin-walled tubes with triangular, square, hexagonal and octagonal sections. Thin-Walled Struct..

[35] San Ha N., Pham T.M., Hao H., Lu G. (2021). Energy absorption characteristics of bio-inspired hierarchical multi-cell square tubes under axial crushing. Int. J. Mech. Sci..

[36] Wu S., Zheng G., Sun G., Liu Q., Li G., Li Q. (2016). On design of multi-cell thin-wall structures for crashworthiness. Int. J. Impact Eng..

[37] Sun G., Deng M., Zheng G., Li Q. (2019). Design for cost performance of crashworthy structures made of high strength steel. Thin-Walled Struct..

[38] Zorzetto L., Ruffoni D. (2017). Re-entrant inclusions in cellular solids: From defects to reinforcements. Compos. Struct..

[39] Qi C., Sun Y., Yang S. (2018). A comparative study on empty and foam-filled hybrid material double-hat beams under lateral impact. Thin-Walled Struct..

[40] Li Q., Wu L., Hu L., Chen T., Zou T., Li E. (2022). Axial compression performance of a bamboo-inspired porous lattice structure. Thin-Walled Struct..

[41] Zhang W., Yin S., Yu T.X., Xu J. (2019). Crushing resistance and energy absorption of pomelo peel inspired hierarchical honeycomb. Int. J. Impact Eng..

[42] Yin H., Wen G., Liu Z., Qing Q. (2014). Crashworthiness optimization design for foam-filled multi-cell thin-walled structures. Thin-Walled Struct..

